# *In vitro* activity of recombinant lysostaphin in combination with linezolid, vancomycin and oxacillin against methicillin-resistant *Staphylococcus aureus*

**Published:** 2017-08

**Authors:** Nagalakshmi Narasimhaswamy, Indira Bairy, Gautham Shenoy, Laxminarayana Bairy

**Affiliations:** 1 Department of Microbiology, Melaka Manipal Medical College (Manipal Campus) Manipal University, Manipal, Karnataka 576104, India; 2 Department of Pharmaceutical Chemistry, Manipal College of Pharmaceutical Sciences, Manipal University, Manipal, Karnataka 576104, India; 3 Department of Pharmacology, RAK Medical and Health Sciences University, Ras Al Khaimah, UAE

**Keywords:** Lysostaphin, Methicillin resistant *Staphylococcus aureus* (MRSA), Synergistic activity

## Abstract

**Background and Objectives:**

The antimicrobial combination with synergistic mechanism is recommended to provide broad-spectrum coverage, and prevent the emergence of resistant mutants. In the present study, the synergistic activity of lysostaphin with linezolid, oxacillin and vancomycin, against methicillin-resistant *Staphylococcus aureus* (MRSA) clinical isolates was determined.

**Materials and Methods:**

Seventy-three MRSA isolates collected from clinical specimens were tested, for *in vitro* synergistic activity of lysostaphin with linezolid, vancomycin and oxacillin, by checkerboard assay.

**Results:**

Lysostaphin showed synergistic activity with linezolid and oxacillin, against all MRSA isolates, tested in the present study. Whereas, only 19.1% of the isolates showed synergistic activity with vancomycin and remaining 80.9% of the MRSA isolates showed additive activity.

**Conclusion:**

Lysostaphin causes rapid lysis of *S. aureus*. Combination therapies that include linezolid and lysostaphin could be used in life-threatening infections, such as endocarditis to increase the early *in vivo* activity of the antibiotics, and to prevent the emergence of linezolid resistant mutants. Further, *in vivo* studies are warranted to confirm our results.

## INTRODUCTION

*S. aureus* is commonly found on the skin and anterior nares of healthy individuals. However, in case of any defect in host immune system, they gain entry into the tissue and cause infections, ranging from the localized abscess to invasive infections, such as skin and soft tissue infections, endocarditis, osteomyelitis, bacteremia and pneumonia. This pathogenicity reflects its ability to produce a variety of exotoxins, and adherence to medical devices, by production of biofilm (1, 2). The development of methicillin resistance, among *S. aureus*, reported in the late 1960s (3). It posed a serious therapeutic challenge and still continues to do so. Currently, MRSA is an important pathogen, associated with both hospital and community-acquired infections. Hospital-acquired MRSA has been a serious problem in health care facilities, worldwide, including India (4). The emergence of multidrug-resistant *S. aureus* has spurred the need for the development of new anti-microbial agents. One such novel bactericidal agent is lysostaphin, which lyses *S. aureus*, through cleaving pentaglycin cross-bridge of the staphylococcal cell wall (5, 6). Though there was a demonstration of the efficacy of lysostaphin, in treating *S. aureus* infections in animal models, the lack of purified preparation of lysostaphin and wide availability of alternative antibiotics, terminated further development of lysostaphin, as an anti-staphylococcal agent. The rapid increase in resistance to available antibiotics and the availability of recombinant lysostaphin, have rekindled the interest, in using lysostaphin as a therapeutic agent for staphylococcal infections (7–9). The use of combinations of antibiotics are recommended, compared to single antibiotic usage in the treatment of severe infections, such as endocarditis (10). The combinations of antibiotics with synergistic mechanism is used to provide broad-spectrum coverage, and to prevent the emergence of resistant mutants (11). In this context, investigations in regards to anti-microbial effects of antibiotics in combination with lysostaphin, is worthwhile. Hence, the aim of the present study was to determine the combination effect of lysostaphin with other anti-microbial agents, against MRSA clinical isolates.

## MATERIALS AND METHODS

### Bacterial strains.

A total of seventy-three clinical samples of MRSA, isolated from various clinical specimens (pus, burn wound swab, blood, urine, sputum, endotracheal aspirate, catheters tips, skin surfaces and nasal swabs), received from Kasturba hospital, Manipal, India, were used for the present study. Methicillin resistance was determined by using Kirby-Bauer disk diffusion method, according to recommendation of the Clinical and Laboratory Standards Institute (12).

### Antimicrobial agents tested.

Recombinant lysostaphin (Sigma Aldrich L9043), vancomycin (Sigma Aldrich 861987), linezolid (Sigma Aldrich PZ0014) and oxacillin (Hi-Media CMS5372-5G), were used in the present study.

### Checkerboard assay.

Synergistic activity of lysostaphin with antibiotics was tested by checkerboard method, as described by Garcia (13). Combination of lysostaphin (0.06 to 4 μg/ml) was tested with oxacillin (1 to 256 μg/ml), vancomycin (0.06 to 8 μg/ml) and linezolid (0.03 to 8 μg/ml). Two-fold dilutions of lysostaphin were prepared in MHB-CA, supplemented with 0.1% BSA, in order to minimize nonspecific binding of lysostaphin to the polystyrene plate. Linezolid, oxacillin and vancomycin dilutions were prepared without BSA. In order to determine the minimum inhibitory concentration (MIC) of individual antibiotic, each concentration was added in the first row and lysostaphin was dispensed in the first column. Further, the combination of each concentration of antibiotic with lysostaphin was dispensed into microtiter plate in a checkerboard style. Wells of the microtiter plate were inoculated with ∼1×10^5^ CFU/ml bacterial cells. The bacterial growth control without the addition of antibiotic was also included. Microtiter plates were incubated at 37°C for 16 hours. The fractional inhibitory concentrations (ΣFIC) indices were calculated, by adding the FICs of drug A (drug A MIC combined/drug A MIC alone) + drug B (drug B MIC combined/drug B MIC alone). Results were interpreted based on ΣFIC as follows: ≤0.5; synergistic, >0.5 and ≤4; additive, >4.0; antagonistic effect. *S. aureus* sub sp. *aureus* ATCC 43300 and ATCC 29213, were used as controls.

## RESULTS

In the present study, lysostaphin was tested for synergistic activity with vancomycin, linezolid and oxacillin. Lysostaphin showed MIC, against ATCC 29213, ATCC 43300 and ATCC 700699 (vancomycin intermediate sensitive *S. aureus*-VISA) at 0.125, 0.5 and 2 μg/ml, respectively. MIC of lysostaphin against MRSA isolates, ranged from 0.25 μg/ml to 2 μg/ml. The MIC_50_ and MIC_90_ are defined, as the MIC that inhibits 50% and 90% of the isolates tested, respectively. Isolates had MIC_50_ and MIC_90_ values of 1 μg/ml to lysostaphin. The range of MIC for oxacillin, linezolid, vancomycin and lysostaphin are shown in Table 1. Eighty-eight percent of isolates had MIC value between 8 to 64 μg/ml to oxacillin, and 12% were highly resistant to oxacillin with MIC of >256 μg/ml. None of the MRSA isolates were resistant to vancomycin and linezolid and their MIC values, ranged from 0.25 μg/ml to 2 μg/ml. MIC_50_ and MIC_90_ were 0.5 and 2 μg/ml, respectively.

**Table 1.** Anti-bacterial activity of lysostaphin, vancomycin, linezolid and oxacillin, tested against MRSA isolates

**Antibacterial agents**	**MIC range (μg/ml)**	**MIC_50_ (μg/ml)**	**MIC_90_ (μg/ml)**	**MBC range (μg/ml)**
Lysostaphin	0.25 to 2	1	1	0.125 to 4
Vancomycin	0.25 to 2	0.5	2	0.5 to 2
Linezolid	0.25 to 2	0.5	2	0.25 to 2
Oxacillin	4 to >256	32	64	8 to 512

**Fig. 1.**
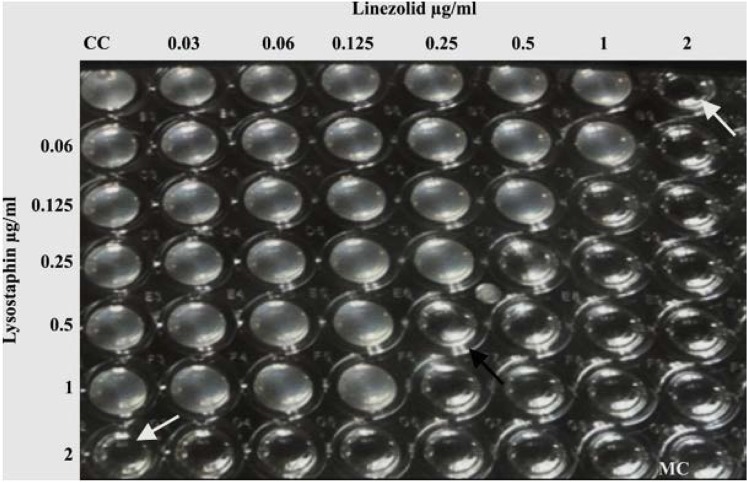
Checkerboard microtiter plate assay: linezolid with lysostaphin combination. The white arrow shows the MIC of drug alone. The black arrow indicates the antibiotic combination that resulted in a synergistic effect; FIC 0.375. CC – cell control, MC – medial control

**Fig. 2.**
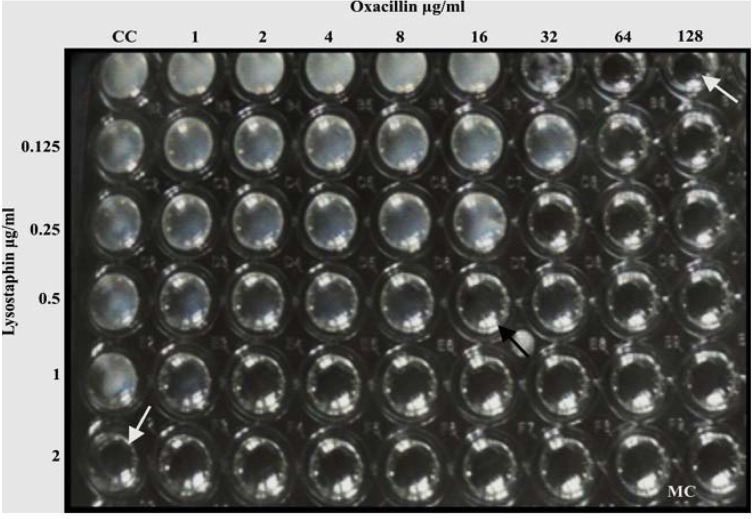
Checkerboard microtiter plate assay: oxacillin with lysostaphin combination. The white arrow shows the MIC of drug alone. The black arrow indicates the antibiotic combination that resulted in a synergistic effect; FIC 0.375. CC – cell control, MC – medial control

**Fig. 3.**
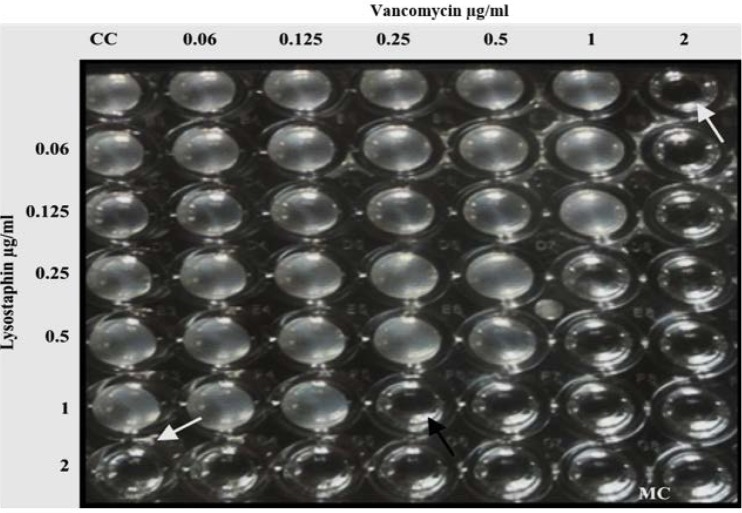
Checkerboard microtiter plate assay: vancomycin with lysostaphin combination. The white arrow shows the MIC of drug alone. The black arrow indicates the antibiotic combination that resulted in a synergistic effect; FIC 0.625. CC- cell control, MC- media control

MRSA clinical isolates and ATCC 700699 were tested for lysostaphin synergistic activity with the individual combination of vancomycin, linezolid and oxacillin. Lysostaphin combined with linezolid as well as oxacillin, showed a synergistic effect, against all the isolates tested in the present study; FIC values ranged from 0.31 to 0.5 ( Figs. 1, 2). Combination of lysostaphin with vancomycin had a FIC values from 0.5 to 0.75 (Fig. 3). According to the FIC values, 0.25 μg/ml of linezolid, combined with 0.5 μg/ml lysostaphin showed synergistic activity against 79.5% of the isolates tested. Remaining 20.5% of the isolates had synergistic activity of the combination of 0.25 μg/ml of linezolid with 1 μg/ml lysostaphin. For oxacillin (16 μg/ml) with lysostaphin (1 μg/ml) combination, the effect was synergistic against 100% of the isolates. Only 19.1% of the isolates showed synergistic activity, when 1 μg/ml lysostaphin combined with 0.5 μg/ml of vancomycin. The combination of 0.5 μg/ml lysostaphin with 0.5 μg/ml of vancomycin found to have additive activity, against remaining 80.9% isolates tested. None of the combination showed an antagonist effect.

## DISCUSSION

The emergence of multidrug-resistant MRSA strains frequently causes treatment failure. The synergistic activity of anti-microbial agents provide broad-spectrum coverage, prevent the development of resistant mutants, and it is considered to be a novel approach for the treatment of systemic life-threatening infections (11, 14). In the present study, synergistic activity of lysostaphin was tested with linezolid, vancomycin and oxacillin against MRSA isolates and VISA (ATCC 700699). Lysostaphin combined with linezolid/oxacillin showed synergistic activity, against both MRSA and VISA; however, with vancomycin only 19.1% of the isolates showed synergistic activity. 80.9% of the MRSA isolates showed additive activity. Lysostaphin combined with linezolid is more active against MRSA/VISA strains, linezolid MIC was found to reduce by sixteen fold with the combination, compared to linezolid alone. As lysostaphin causes rapid lysis of bacterial cells, combination therapies that include linezolid and lysostaphin could be used in life-threatening infections such an endocarditis, to increase the early *in vivo* activity of the drugs and to prevent the emergence of linezolid/vancomycin resistant mutants. In the present study, all the MRSA isolates showed synergistic activity with oxacillin, when combined with lysostaphin. Earlier studies have reported that lysostaphin-resistant staphylococcal variants become more susceptible to β-lactam antibiotics, compared to their parental strains. Resistance to lysostaphin among *S. aureus* is due to changes in the muropeptide cross-bridge. Mutations in *femA*, which controls the addition of the second and third glycines of the forming cross-bridge, result in the formation of a new cross-bridge structure, composed of a single glycine. Strains with monoglycine cross-bridge are found to be lysostaphin resistant, but also become hyper-susceptible to β-lactam antibiotics. This mechanism of resistance gives partial explanation, regarding the observed synergism, among *S. aureus* strains treated with combinations of lysostaphin and β-lactams (15, 16). In order to suppress the emergence of lysostaphin resistant mutants, the combination of β-lactam drug could be a better choice. Previous reports showed bactericidal synergy of lysostaphin with bacitracin, polymixin B, daptomycin and additive effect with gentamycin, tetracycline and erythromycin (17, 18). Kiri et al. reported, *in vivo* synergistic activity of lysostaphin with nafcillin, reduced the dose of lysostaphin to 1 mg/kg to treat systemic infection caused by *S. aureus* in mouse (19). The additive effect of lysostaphin and vancomycin was demonstrated in *S. aureus* infective endocarditis model (20, 21). Climo et al. reported that intravenous administration of lysostaphin (weekly; 15mg/kg) for nine weeks had excellent bactericidal activity irrespective of the formation of neutralizing antibodies. They also observed that lysostaphin was well tolerated during long-term dosing, without any hypersensitivity reactions (20). Sei et al. observed that lysostaphin able to prevent *S. aureus* induced systemic shock by blunting the inflammatory cytokine mediated response, which results in reduced expression of TNF and IL-6 (22). These findings are supported with Ip et al. demonstrated that lysostaphin rapidly lyses all free bacteria, thus preventing their uptake in phagosomes, as whole bacteria need to be engulfed by phagosomes to induce TLR-dependent cytokine production (23). In the present study, lysostaphin combined with linezolid was able to kill VISA strain and this synergistic effect could be used, as an alternative therapeutic choice to treat infection, caused by vancomycin resistant *S. aureus*. Lysostaphin showed promising activity, against MRSA and VISA isolates; no resistance was found among them. Further study, on *in vivo* synergistic activity is recommended, before taking up clinical studies.

## References

[1] Lowy FD. *Staphylococcus aureus* infections. N Engl J Med 1998;339:520–532. 9709046 10.1056/NEJM199808203390806

[2] Ryu S Song PI Seo CH Cheong H Park Y. Colonization and infection of the skin by *S. aureus*: immune system evasion and the response to cationic antimicrobial peptides. Int J Mol Sci 2014;15:8753–8772. 24840573 10.3390/ijms15058753 PMC4057757

[3] Jevons MP. “Celbenin”-resistant *Staphylococci*. Brit Med J 1961;1(5219):124–125.

[4] Grundmann H Aires-de-Sousa M Boyce J Tiemersma E. Emergence and resurgence of meticillin-resistant *Staphylococcus aureus* as a public-health threat. Lancet 2006;368(9538):874–885. 16950365 10.1016/S0140-6736(06)68853-3

[5] Gründling A Schneewind O. Cross-linked peptidoglycan mediates lysostaphin binding to the cell wall envelope of *Staphylococcus aureus*. J Bacteriol 2006;188:2463–2472. 16547033 10.1128/JB.188.7.2463-2472.2006 PMC1428428

[6] Browder HP Zygmunt WA Young JR Tavormina PA. Lysostaphin: enzymatic mode of action. Biochem Biophys Res Commun 1965;19:383–389. 14317407 10.1016/0006-291x(65)90473-0

[7] Kumar A Khan IA Sharma PR Sumathy K Krishna ME. Evaluation of activity of recombinant lysostaphin against isolates of meticillin-resistant *Staphylococcus aureus* from Indian hospitals. J Med Microbiol 2014;63:763–766. 24623634 10.1099/jmm.0.070557-0

[8] Kumar JK. Lysostaphin: an antistaphylococcal agent. Appl Microbiol Biotechnol 2008;80:555–561. 18607587 10.1007/s00253-008-1579-y

[9] Kokai-Kun J (2012). Lysostaphin: a silver bullet for staph. In: Tegos A. M E, ed, Antimicrobial Drug Discovery. CABI publishing, 1st ed Oxfordshire, Walling-ford, UK, pp. 147–165.

[10] Grohs P Kitzis MD Gutmann L *In vitro* bactericidal activities of linezolid in combination with vancomycin, gentamicin, ciprofloxacin, fusidic acid, and rifampin against *Staphylococcus aureus*. Antimicrob Agents Chemother 2003;47:418–420. 12499229 10.1128/AAC.47.1.418-420.2003 PMC148978

[11] Grif K Dierich MP Pfaller K Miglioli PA Allerberger F. *In vitro* activity of fosfomycin in combination with various antistaphylococcal substances. J Antimicrob Chemother 2001;48:209–217. 11481290 10.1093/jac/48.2.209

[12] CLSI (2010). Performance Standards for Antimicrobial Susceptibility Testing: Twentieth Informational Supplement. M100–S20. PA, USA.

[13] Garcia LS (2010). Clinical Microbiology Procedures Handbook. 3rd ed American Society of Microbiology Press Washington DC.

[14] Jacqueline C Caillon J Le Mabecque V Miegeville AF Donnio PY Bugnon D *In vitro* activity of linezolid alone and in combination with gentamicin, vancomycin or rifampicin against methicillin-resistant *Staphylococcus aureus* by time-kill curve methods. J Antimicrob Chemother 2003;51:857–864. 12654769 10.1093/jac/dkg160

[15] Climo MW Ehlert K Archer GL. Mechanism and suppression of lysostaphin resistance in oxacillin-resistant *Staphylococcus aureus*. Antimicrob Agents Chemother 2001;45:1431–1437. 11302806 10.1128/AAC.45.5.1431-1437.2001 PMC90484

[16] Kusuma C Jadanova A Chanturiya T Kokai-Kun JF. Lysostaphin-resistant variants of *Staphylococcus aureus* demonstrate reduced fitness *in vitro* and *in vivo*. Antimicrob Agents Chemother 2007;51:475–482. 17101683 10.1128/AAC.00786-06 PMC1797764

[17] Polak J Latta PD Blackburn P. *In vitro* activity of recombinant lysostaphin-antibiotic combinations toward methicillin-resistant *Staphylococcus aureus*. Diagn Microbiol Infect Dis 1993;17:265–270. 8112040 10.1016/0732-8893(93)90034-5

[18] Desbois AP Coote PJ. Bactericidal synergy of lysostaphin in combination with antimicrobial peptides. Eur J Clin Microbiol Infect Dis 2011;30:1015–1021. 21311938 10.1007/s10096-011-1188-z

[19] Kiri N Archer G Climo MW. Combinations of lysostaphin with β-lactams are synergistic against oxacillin-resistant *Staphylococcus epidermidis*. Antimicrob Agents Chemother 2002;46:2017–2020. 12019130 10.1128/AAC.46.6.2017-2020.2002 PMC127219

[20] Climo MW Patron RL Goldstein BP Archer GL. Lysostaphin treatment of experimental methicillin-resistant *Staphylococcus aureus* aortic valve endocarditis. Antimicrob Agents Chemother 1998;42:1355–1360. 9624475 10.1128/aac.42.6.1355 PMC105603

[21] Kokai-Kun JF Chanturiya T Mond JJ. Lysostaphin as a treatment for systemic *Staphylococcus aureus* infection in a mouse model. J Antimicrob Chemother 2007;60:1051–1059. 17848374 10.1093/jac/dkm347

[22] Clara Sei TC Mond James J. Kokai-Kun John F. Lysostaphin reduces the production of inflammatory cytokines in *Staphylococcus aureus* challenged mice, and prevents systemic shock. Open Antimicrobl Agents J 2011;3:6–11.

[23] Ip WK Sokolovska A Charriere GM Boyer L Dejardin S Cappillino MP Phagocytosis and phagosome acidification are required for pathogen processing and MyD88-dependent responses to *Staphylococcus aureus*. J Immunol 2010;184:7071–7081. 20483752 10.4049/jimmunol.1000110 PMC2935932

